# A Cohort Study on the Impact of Oral Health on the Quality of Life of Adolescents and Young Adults

**DOI:** 10.3390/clinpract15040076

**Published:** 2025-04-07

**Authors:** Iva Klarić Puđa, Kristina Goršeta, Hrvoje Jurić, Mirko Soldo, Luc A. M. Marks, Martina Majstorović

**Affiliations:** 1 Health Center—Zagreb Center, Runjaninova 4, 10000 Zagreb, Croatia; 2 Department of Paediatric Dentistry, School of Dental Medicine, University of Zagreb, Gunduliceva 5, 10000 Zagreb, Croatia; gorseta@sfzg.hr (K.G.); juric@sfzg.hr (H.J.); 3 Community Health Center Osječko-Baranjska County, Park kralja Petra Krešimira IV 6, 31000 Osijek, Croatia; ortodoncija1.osijek@dzobz.hr; 4 Faculty of Medicine & Health Sciences, University of Antwerp, 2610 Antwerp, Belgium; luc.marks@uza.be; 5 Special Care in Dentistry, Department of Cranio-Maxillofacial Surgery, Antwerp University Hospital, 2650 Edegem, Belgium; 6 Department of Orthodontics and Pediatric Dentistry, University of Maryland Baltimore School of Dentistry, Baltimore, MD 21202, USA; mmajstorovic@umaryland.edu

**Keywords:** adolescents, young adults, oral health, quality of life, DMFT

## Abstract

**Objectives:** This cohort study examines the relationship between quality of life and oral health in adolescents and young adults in the Zagreb area. **Methods:** The research involved 250 participants aged 14 to 25 from Zagreb. Each participant was examined by an oral medicine doctor using a probe and mirror in a dental unit, and their DMFT (Decayed, Missing, and Filled Teeth) status was determined. Participants also completed questionnaires on their socio-economic status (SES) and the impact of their oral health on quality of life (OHIP-14, Oral Health Impact Profile). **Results:** Caries was the most common dental issue among adolescents (2.23 ± 2.58), with restoration being the most frequent treatment (54%). Endodontic treatment and tooth extraction were more prevalent among individuals with lower SES (24.1%), who also had worse DMFT scores (8.09 ± 5.56). Prophylaxis was equally distributed across SES and gender. Male patients had more carious teeth (2.75 ± 3.07) than female patients (1.85 ± 2.08), while female patients scored worse on the OHIP-14 scale (10.97 ± 8.77) compared to males (8.81 ± 8.11). Age positively correlated with both OHIP-14 and DMFT scores. **Conclusions:** Adolescents and young adults in Zagreb, Croatia, exhibited high DMFT and OHIP-14 scores, reflecting significant oral health issues and reduced quality of life, particularly among older individuals and those with lower SES. The association between invasive treatments (e.g., endodontic procedures and extractions) and diminished quality of life underscores the necessity for early preventive measures, including regular dental check-ups and targeted oral health education.

## 1. Introduction

The World Health Organization (WHO) defines health as “a state of complete physical, mental, and social well-being, and not merely the absence of disease or infirmity” [1]. The aspect of “social well-being” is often overlooked when considering health. According to WHO, an individual’s quality of life (QoL) is their perception of their position in life within the context of the cultural and value systems in which they live, and in relation to their goals, expectations, standards, and concerns. Quality of life is influenced by physical health, mental status, level of independence, social relationships, and personal attitudes toward the environment [2,3,4].

Oral health is multifaceted, encompassing the ability to speak, laugh, smell, taste, touch, chew, swallow, and express a range of emotions through facial expressions confidently and without pain, discomfort, or disorders of the craniofacial complex (head, face, and oral caries) [3,4,5].

Regardless of age, oral health is critical to overall health and well-being [3,5]. Oral diseases affect both individual and population well-being. From birth, the maxillofacial system plays a key role in the physical, psychological, and social aspects of a person’s life [6]. According to WHO, oral diseases, especially caries and periodontal diseases, are among the most common diseases worldwide [7].

While the situation regarding dental caries has improved in recent decades, the number of dental visits among adolescents remains stagnant. Periodontitis is less common among adolescents, but plaque, gingivitis, and poor oral hygiene are widespread, particularly among males [7,8]. Dietary habits have shifted, with young people increasingly consuming processed foods high in sugar, refined carbohydrates, and fats, leading to more carious teeth and worse DMFT (Decayed, Missing, and Filled Teeth) status [8].

Adolescence is a period of significant change, where individuals transition from dependence on parents or guardians to making their own decisions [9]. It is a time of biological, emotional, and social changes, where individuals become independent and take responsibility for their health [10]. The influence of society and socio-economic status (SES) in early life on oral health persists throughout life. Adolescents from lower socio-economic backgrounds are at higher risk of infections, smoking, and poor oral hygiene, all of which increase the risk of oral diseases [11]. Quality of life, including perceptions of oral health, tooth loss, aesthetics, and general appearance have a substantial impact on young adults, and are particularly challenging in adolescence. Common issues reported by adolescents include diet, smile, halitosis, and mild pain [12].

Poor oral health in adolescence leads to poor oral status and lower quality of life later in life [13]. Regular dental visits are associated with better subjective assessments of oral health [11]. To determine the impact of oral health on overall quality of life, specific parameters are needed [14].

The primary goal of healthcare is to cure disease, extend life expectancy, improve overall quality of life, and achieve a satisfactory level of well-being during the most active and productive periods of life [15]. Understanding oral health-related quality of life (OHRQoL) better can contribute to developing strategies aimed at improving health education and prevention [2].

The aim of this study is to determine the influence of oral health on the quality of life of adolescents and young adults. To date, there are no similar studies in Croatia and only a few in the European Union [16,17,18,19] that specifically address this problem in the targeted age group. Therefore, the goal is also to acknowledge the need for such and similar studies in culturally and geographically diverse populations, based on their specific needs and expectations.

Although previous studies have explored OHRQoL among adolescents in various regions, the present study is distinctive in its focus on a previously underrepresented population, namely, adolescents and young adults in Croatia. To date, there are limited data available from Croatia and few comparable studies from the wider region specifically examining the relationship between SES, detailed treatment modalities, and QoL indicators in this age group.

## 2. Materials and Methods

This study was designed as a cohort study, assessing the relationship between oral health status and quality of life among adolescents and young adults. The participants, aged 14 to 25, were randomly selected from the Zagreb area. Participants were recruited using convenience sampling at three clinical locations in Zagreb (a private dispensary, the School of Dental Medicine, and the Medical Center Zagreb—Center). Recruitment took place over a 12-month period, from 1 June 2020 to 1 June 2021. The inclusion criteria were as follows: (1) participants aged between 14 and 25 years, (2) permanent residence in the Zagreb area, (3) willingness and capacity to provide informed consent (or parental consent for participants younger than 18 years), and (4) absence of acute dental pain or emergencies at the time of recruitment. The exclusion criteria were the following: (1) individuals outside the specified age range, (2) refusal or inability to provide informed consent, (3) presence of severe medical or psychological conditions impacting oral health or the participant’s ability to complete questionnaires, and (4) ongoing orthodontic treatment. Participants meeting the criteria were informed about the study’s objectives and procedures, after which they provided written informed consent. A total of 250 participants took part in the study: 105 males (42%) and 145 females (58%). The average age of all participants was 19.7 years (SD = 3.58).

All intraoral examinations were performed by a single doctor of dental medicine using a probe and mirror in a dental office, and the DMFT status of each participant was determined.

Conducting the study across three different locations allowed for a diverse and representative sample, incorporating variations in socio-economic, cultural, and environmental factors that may influence oral health. While this approach enhances the generalizability of findings, differences in healthcare accessibility and attitudes toward dental care across locations should be considered when interpreting the results. Variations in healthcare accessibility, affordability, and the availability of preventive dental services across study locations may influence oral health outcomes. Such factors could introduce differences in treatment-seeking behavior, oral hygiene habits, and overall dental care utilization, reflecting local influences rather than a uniform trend across the entire population studied. Although data were collected at three locations to ensure a diverse sample, the study was designed to assess overall trends rather than to compare outcomes across sites.

The questionnaires used in this research are detailed in the subsections below.

### 2.1. Oral Health Impact Profile (OHIP-14) Questionnaire

The Croatian version, translated according to accepted professional methods, was used. The original questionnaire consists of 49 questions, but a shortened version is most commonly used [20]. The Oral Health Impact Profile (OHIP-14) is one of the questionnaires evaluated to determine how quality of life depends on oral health. It contains 14 questions about oral health [21]. The OHIP-14 questionnaire, originally developed by Slade and Spencer (1994) for measuring disability and discomfort due to oral conditions, is one of the most widely known OHRQoL instruments. It consists of 14 items derived from the original 49-item version. The OHIP-14 questionnaire used in this study examines the level of oral health across 7 domains: functional limitations, physical pain, psychological discomfort, physical disability, psychological disability, social disability, and handicap. Answers are rated on a scale from 0 to 4: 0—never, 1—very rarely, 2—occasionally, 3—often, and 4—very often. The highest possible score is 56, with a higher total score indicating poorer life quality in relation to oral health [22,23].

### 2.2. Socio-Economic Status (SES) Questionnaire

The SES scale values were determined using the questionnaire outlined in Table 1. Given the many variations of the SES questionnaire, this research limited the questions to a minimum number commonly found in most questionnaires. The SES indicator is a sum of points from all 11 questions. To enhance precision, the total score is categorized into three levels: 1—low, 2—middle, and 3—high. Life quality was estimated using one of the evaluated methods for determining life quality in relation to oral health, as shown in Table 1.

### 2.3. DMFT

DMFT is the sum of decayed, missing due to caries, and filled teeth with respect to permanent teeth. The mean DMFT is the sum of individual DMFT values divided by the population total. The DMFT index is a widely used epidemiological measure that reflects an individual’s cumulative experience with dental caries by accounting for teeth that are currently decayed, have been extracted due to caries, or have been restored [24,25]. The DMFT index was determined through intraoral examination using a probe and mirror. The analysis includes total DMFT and OHIP-14 scores, age and gender of participants, SES categorized into three levels (low, medium, and high), and the last dental treatment received, categorized into three types: 1—prophylaxis, 2—restoration, 3—endodontics and extraction.

### 2.4. Statistical Analysis

The necessary sample size was estimated using the most demanding method applied to test the hypotheses: the χ^2^ test. Using GPower 3.1 software, the minimum sample size was calculated to be 220 participants, assuming a medium effect size (0.3), an alpha error probability (α) of 0.05, a power (1−β error probability) of 0.95, and 5 degrees of freedom. In addition to describing individual research variables, the following methods were used to test the hypotheses. Nominal variables (gender) and scales (SES and applied treatment) are described by frequencies, and their relationships were tested using the χ^2^ test. Continuous variables (DMFT and OHIP-14) were tested for normality using the Kolmogorov–Smirnov test and described by mean and standard deviation. The reliability of the OHIP-14 scale was checked by Cronbach’s alpha coefficients. The interdependence of age, DMFT, and OHIP-14 was tested with the Pearson correlation coefficient. Differences among subgroups, determined by gender, SES categories, and treatment types, were tested using the *t*-test for independent samples and one-way analysis of variance. The default significance level was set to 0.05. STATISTICA version 10 and SPSS 18 were used for data processing and analysis.

## 3. Results

### 3.1. Socio-Economic Status

The SES of the respondents was distributed as follows: low (23.2%), medium (55.6%), and high (21.2%). The test results and corresponding contingency table are presented in Table 2.

### 3.2. Dental Treatment

The frequency of dental treatments did not differ by gender. Prophylaxis was performed on 29.6% of respondents (31.4% of males and 28.3% of females). Restorative treatments were the most common, applied to 54% of respondents (52.4% of males and 55.2% of females). Endodontics and extractions were performed on 16.4% of respondents (16.2% of males and 16.6% of females). The distribution of treatment categories indicates that the applied treatments were similar across genders, as confirmed by the test results in Table 3.

The distribution of treatment categories was consistent across all SES groups. As shown in Table 4, restorative treatments were slightly less common among respondents with low SES (46.6%) and slightly more common among those with high SES (66%) compared to the overall share of 54%. Endodontics and extractions were more prevalent among respondents with low SES (23.2%). However, these differences were not statistically significant, with a probability of independence of 0.168. The application of prophylaxis was similar across SES categories, at 29.8% of all respondents (Table 4).

### 3.3. Oral Health Status

Oral health status, including the number of healthy, extracted, filled, and carious teeth per person, and the DMFT index, did not differ significantly by gender, except for caries. Males had an average of 2.76 carious teeth per person, while females had significantly fewer, at 1.85. This difference did not result in significant differences in the DMFT index by gender. The average values of the total number of individual categories of teeth are listed in Table 5.

The number of teeth per person did not differ significantly by SES. Therefore, Table 6 lists the test results for the DMFT index only. On average, respondents with low SES had nearly two more DMFT teeth than those with higher status, but this difference was only marginally significant, with an 8% error margin.

The type of dental treatment significantly affected the number of healthy, carious, extracted, and filled teeth per person, as well as the DMFT index. The number of healthy teeth per person decreased significantly across treatment categories in the order of prophylaxis, restoration, and endodontics and extraction. Conversely, the number of carious, extracted, and filled teeth per person, as well as the DMFT index, increased significantly across these categories (Table 7).

### 3.4. Quality of Life

The reliability and internal consistency of the OHIP-14 questionnaire were satisfactory, with a Cronbach’s α coefficient of 0.878, well above the minimum required of 0.7. Internal consistency was confirmed by Cronbach’s α coefficients, even when individual items were omitted, resulting in similar coefficients to the complete questionnaire. As shown in Table 8, the average OHIP-14 scores did not differ significantly by SES categories. However, significant differences were observed by gender (*p* = 0.049) and treatment type (*p* < 0.001), with scores increasing in the order of prophylaxis, restoration, and endodontics and extraction. The Welch test confirmed the OHIP-14 results by gender and treatment but nullified the significance of differences for SES with an 8% error margin.

The age of respondents was significantly correlated with both the DMFT index and OHIP-14 scores. Both indices showed a significant positive correlation with age. The DMFT index increased significantly with age. For respondents aged 14 to 20 years, the average DMFT was 5.19 teeth (SD = 3.71), while for those aged 21 to 25 years, it was 8.90 teeth (SD = 5.52). This difference was statistically significant (*t* = −6.328, df = 248, *p* < 0.001). According to regression analysis, the DMFT index increased by 0.782 per year for males and 0.496 per year for females.

## 4. Discussion

The results indicate that the distribution of treatments during dental visits does not differ by gender: prophylaxis (31.4% male, 28.3% female), restoration (52.4% male, 55.2% female), and endodontics/extraction (16.2% male, 16.6% female). This distribution reflects current clinical demand in the population, where restorative and surgical interventions (albeit sometimes scheduled as follow-ups to routine examinations) are recorded more frequently than purely preventive interventions. Hence, our results do not necessarily indicate a lack of preventive focus in dental services, but rather may reflect a combination of treatment needs and service utilization behaviors within the system. The DMFT status of respondents is 6.87 ± 4.9, with no statistically significant difference between genders. Males have more carious teeth (2.75 ± 3.07) compared to females (1.85 ± 2.08).

A 2014 study in a similar age group (16–25) in Mexico shows a lower DMFT index (4.24 ± 3.85). In contrast to our results, females in that study had a greater propensity for caries, while the components of extracted and filled teeth were equal in both genders [26]. A 2022 study in Kosovo found that 15-year-old boys had a higher number of decayed teeth, while the number of filled and missing teeth was approximately the same among genders [27].

A 2018 study on adolescents (15–24 years) in Uruguay shows a significantly lower DMFT index (3.60 ± 1.36) compared to our study [28]. Post-adolescents (18–25 years) in Russia (Drachev et al., 2017) had a slightly higher DMFT index (7.58 ± 0.61) than our study [29]. Also, in the study from Russia, SES was not significantly associated with DMFT. To better interpret the observed differences in DMFT values across countries, it is important to consider the organization and accessibility of dental care. In the Russian cohort studied by Drachev et al. [29], most students were eligible for free education, and the dominant FT (“Filled Teeth”) component of the DMFT index (6.84 out of 7.58) indicates a high rate of restorative treatment, possibly facilitated by accessible, subsidized care within university settings. While the study by Goettems et al. [28] explicitly states that orthodontic treatment is not covered by the Uruguayan public health system, no information is provided on the coverage of other dental services. Therefore, it is not possible to draw conclusions about the extent of public funding for preventive or restorative treatments at the specific time and locations in which the study was conducted. Results from our study show that the DMFT in young people aged 21 to 25 is higher by 3.71 compared to those aged 14 to 20, indicating how rapidly DMFT increases with age. Consistent with other findings, the DMFT index in this study increased with age, as dental caries is an irreversible, accumulative disease.

From Table 8, we see that DMFT and OHIP-14 positively correlate with age and with each other. Based on the results, we can conclude that oral health affects the subjective perception of quality of life. García-Cortés et al. (2014) found that the number of filled teeth is proportional to age; however, their study did not establish a direct relationship between dental health and subjective perception of quality of life [26]. Drachev et al. (2017) also confirmed that DMFT correlates with age. Respondents aged 21 to 25 had a 1.09 higher DMFT than those aged 18 to 20, and females had a higher DMFT [29].

Respondents in Uruguay (Goettems, 2018) with poorer SES and education showed poorer DMFT and higher tooth decay, similar to our results listed in Table 4 [28]. The OHIP-14 scores correlate positively with the complexity of the treatment required. Subjects who underwent prophylactic procedures such as scaling or polishing had the lowest OHIP-14 score (7.53 ± 6.74). Patients requiring restorative treatment had a slightly higher average OHIP-14 value (9.21 ± 7.91). Those needing more demanding treatments, such as endodontics or tooth extraction, had the worst scores (17.44 ± 10.21). Patients receiving more demanding treatment also showed worse DMFT results. The association of higher OHIP-14 scores and DMFT with more complex treatments such as endodontics or tooth extraction probably reflects a higher cumulative burden of oral disease at the time of treatment. It should be noted that such procedures are often scheduled as part of routine care or follow-up after preventive examinations and do not necessarily indicate delayed treatment or poor oral hygiene behavior.

Results from Hong Kong show that the impact of oral health on quality of life was low in 18-year-olds, with a very low DMFT index (1.4) [30]. Oscarson (2007) found that young people in Sweden scored well on quality of life and oral health, with little difference between high and low caries risk groups [31]. Papaioannou et al. (2011) reported significantly lower OHIP-14 scores (1.24 ± 2.04) in adolescents (15–18 years), with no gender difference [32]. Although their age range was lower, the results suggest worse outcomes among Croatian adolescents, where the DMFT for individuals aged 14 to 18 was 5.19 ± 3.71. Colussi et al. (2017) reported OHIP-14 scores of 7.25 ± 6.78 among Brazilian respondents (15–19 years), better than our adolescents and post-adolescents (10.06 ± 8.55) [12].

The findings reveal interesting gender differences in oral health outcomes. While males had a higher prevalence of tooth decay, females reported worse quality of life related to oral health, possibly due to cultural factors. This contrasts with some studies, such as that of Sun et al. (2018), which found that females had more tooth decay but no difference in quality of life outcomes by gender [9]. These findings highlight the need for gender-specific public health strategies addressing both clinical and psychosocial aspects of oral health.

The study underscores a correlation between oral health status and quality of life, with more severe dental conditions leading to higher OHIP-14 scores. This relationship is evident across various studies, including those by Colussi (2017) and Oscarson (2007), although the severity of impact differs [12,31]. The results suggest that more complex dental treatments, such as endodontics and extractions, are associated with worse quality of life outcomes, emphasizing the importance of early preventive measures to avoid invasive procedures. Dental care policies should focus on enhancing preventive services to maintain better overall health and QoL.

Oral health and overall QoL are closely interconnected and influence each other in complex ways. This is true across the lifespan, which is why WHO recognizes oral health as an integral part of overall well-being [33]. Adolescence and young adulthood are critical periods in which oral health can have a significant impact on quality of life and vice versa. For example, oral health problems can affect basic functions such as eating, sleeping, and socializing, as well as undermine self-esteem [33]. Conversely, aspects of QoL such as heightened stress levels or other mental health issues can affect oral behavior and outcomes [34]. Poor oral health often diminishes daily QoL of adolescents and young adults. Dental pain and disease can impair school performance, nutrition, and social confidence. Longitudinal studies show that even a single episode of dental pain in early adolescence can have lasting negative effects on OHRQoL [35]. Similarly, adolescents with more untreated caries or gingivitis report greater physical discomfort, as well as social and emotional problems. One cohort found that adolescents with a higher number of decayed or filled teeth and poorer self-reported oral health had significantly lower levels of happiness, and their happiness levels tended to worsen over time [36]. Cosmetic problems due to impaired oral health can also lead to embarrassment and social avoidance, further affecting psychological well-being. These patterns are also observed in age groups beyond adolescence and young adult; for example, older adults who retain all or most of their natural teeth report higher levels of life satisfaction than those with extensive tooth loss [37].

QoL also affects oral health, particularly through psychological and behavioral factors. Adolescents who experience psychosocial stress or have low life satisfaction are less likely to maintain good oral hygiene habits. This was demonstrated in a large school-based study, which showed that stressed and unhappy adolescents were significantly less likely to brush their teeth regularly on a daily basis, increasing their risk for caries and gingivitis [34]. A study in adults similarly indicates that depression and poor mental health are associated with poorer oral health outcomes, including higher rates of tooth decay and periodontal disease [38]. Socio-economic challenges, as a specific component of QoL, further exacerbate this cycle; for example, adolescents from disadvantaged backgrounds tend to have both poorer oral health and lower OHRQoL [39]. Low QoL environments may promote behaviors (such as smoking, sugary diets, or inadequate hygiene) and stress responses that undermine oral health, while, conversely, good QoL supports positive health behaviors. Overall, the research highlights a bidirectional relationship in which good oral health supports a better quality of life, and a positive, healthy living environment in turn promotes better oral health. This is especially pronounced for adolescents and young adults, where addressing oral health can have far-reaching psychosocial benefits [35].

Oral health status can be influenced by personal, local, and social factors. Significant differences were found between urban and rural areas. The average DMFT index among Croatian adolescents and youth is 6.87 ± 4.97, higher than in Hong Kong (1.92 ± 2.37), Mexico (4.24 ± 3.85), Kosovo (3.21 ± 2.19), and Uruguay (3.6 ± 1.36) [9,26,27,28]. However, respondents in northwestern Russia (18–25 years) had slightly worse results, with an average DMFT of 7.58 ± 0.61 [29]. The OHIP-14 results among Croatian adolescents and young people (10.06 ± 8.55) are worse than those in other regions.

Differences in oral health outcomes compared to other countries may reflect variations in cultural practices, healthcare system variations, and socio-economic conditions. Understanding these factors may help interpret results and tailor public health interventions to address oral health disparities effectively [9,21].

Cultural norms and practices related to diet, oral hygiene, and attitudes toward dental care may influence oral health outcomes. In some cultures, diets high in sugar and carbohydrates or traditional practices that do not prioritize oral hygiene contribute to higher rates of tooth decay and poor DMFT scores. Cultural attitudes toward dental visits might also contribute to less frequent preventive care, leading to worse oral health outcomes [2,3,6,10].

Healthcare systems and their accessibility play a crucial role in determining oral health outcomes. Countries with universal healthcare that includes comprehensive dental coverage tend to have better overall oral health. Conversely, in countries where dental care is less accessible or affordable, individuals from lower socio-economic backgrounds may have limited access to preventive services, resulting in poorer outcomes [1,11].

Socio-economic disparities are often an important factor influencing oral health. Countries with significant income inequality may see more pronounced differences in oral health outcomes between socio-economic groups. In contrast, countries with more equitable wealth distribution and better social safety nets might exhibit smaller disparities. These socio-economic factors, coupled with access to education and resources, may influence the effectiveness of oral health interventions and overall population health [8,28,40].

Although the specific contributions of these factors vary widely from country to country and are not always well documented, the existing studies nevertheless provide a useful context. The Russian study [29] pointed to a paradoxical relationship where SES positively correlated with increased sugar consumption and consequently higher DMFT scores, alongside generally good but irregular dental hygiene and a high utilization of restorative dental care. Similarly, the Kosovo study [27] explicitly identified inadequate oral hygiene (brushing once a day) and indicated insufficient preventive dental services. The Uruguayan study [28] focused exclusively on the need for orthodontic treatment and emphasized that orthodontic treatment is generally not covered by public health services. The Mexican study [26] made an explicit link between socio-economic disadvantage and unmet need for dental care, but did not include detailed information on specific dietary, hygiene, or prevention patterns. Unfortunately, no explicit data on diet, hygiene practices, dental care utilization, health systems, or socio-economic inequalities were available in the Hong Kong study [30]. Given the variability and limitations of these results, broad generalizations or comparative conclusions between Croatia and these countries are not possible.

Methodological differences in study design, data collection, and population sampling can also contribute to discrepancies between studies. Variations in age groups, assessment criteria, and study timing can lead to differing results. Public health initiatives and policies in place at the time of the studies can also influence outcomes, making it crucial to consider the local context when comparing results [15,21,22].

SES plays a crucial role in determining oral health outcomes. This study found that individuals from lower socio-economic backgrounds exhibited poorer DMFT scores and higher levels of tooth decay, consistent with global findings. For example, Goettems (2018) observed similar trends in Uruguay, where poorer education and SES were linked to worse oral health outcomes. These patterns underscore the significant impact of socio-economic disparities on access to preventive care and treatment, leading to poorer oral health. Public health interventions must prioritize reducing these disparities by improving access to affordable dental care and education for lower income populations [28,40,41]. Chaffee et al. discovered that subjective quality-of-life measures can vary depending on social contexts, impacting service utilization, assessment of oral health interventions, and measurement of disease morbidity in low-SES populations [42].

The findings of this study may have implications for oral health policy, public health interventions, and clinical practice, as they indicate a statistically significant correlation between lower SES and poorer oral health-related quality of life. Although the trend in DMFT scores across SES categories followed the expected direction, it did not reach statistical significance. Therefore, these associations should be interpreted with caution, given the study design, convenience sampling, and other limitations.

However, it is important to acknowledge the limitations of this study. Its cross-sectional design limits the ability to infer causality, and potential biases, such as self-reported data and selection bias, may influence the results. The DMFT index was determined through intraoral examination using a probe and mirror, following standard WHO criteria for epidemiological studies [43]. While this method provides a practical and widely accepted means of assessing caries experience, it may have limitations in detecting initial carious lesions compared to additional diagnostic tools such as radiographs [44]. Despite these limitations, the study’s findings contribute valuable insights into the relationship between SES and oral health, reinforcing the need for targeted public health interventions.

Another limitation of this study is that it relies on adolescents’ self-reported SES [45,46] and health [47,48] perceptions. Younger adolescents may have difficulty accurately reporting SES due to factors such as cognitive maturity, limited knowledge of household economics, and even the social desirability bias [49]. Previous studies have shown that adolescents’ perceptions of family socio-economic conditions may differ from objectively measured socio-economic indicators [50,51]. Furthermore, self-perceived health status at a young age may reflect subjective feelings rather than objective health indicators [5,15]. It has also been reported that adolescents’ self-reported SES significantly influences their perceived health, sometimes more so than objective SES indicators, raising concerns about the reliability of self-reports [47]. Another study found moderate stability in adolescents’ self-rated health over time, but also found discrepancies between self-reports and parental ratings, particularly for poorer health, suggesting possible limitations in self-reported health data [48]. Despite these multiple limitations, adolescent self-reports remain valuable as they reflect personal experiences and perceptions that shape their health-related behavior [52].

While this study focused on adolescents and young adults, it is important to point out the importance of prevention strategies that begin earlier in life. For example, a longitudinal study found that the prevalence of caries at the age of six was associated with significantly lower quality of life at the age of ten, emphasizing the need to start preventive measures at a young age [53]. Another study found that children who had their first preventive dental visit before the age of two required fewer operative treatments later in life than children whose first visit was after infancy [54]. Early educational and preventive interventions were shown to improve oral health outcomes in adolescence and adulthood [55,56]. One possible approach includes dental programs in schools [57], which can work in a variety of ways: through oral health education, the application of fluoride varnishes or sealants, and on-site dental screenings. Such programs can improve children’s oral hygiene practices and reduce the incidence of caries. However, a recent survey of European Union countries showed that access to such programs varies widely, with many countries not routinely providing free pre-school dental check-ups, and public insurance often allowing greater coverage for treatment of existing conditions rather than for preventive services [58]. These findings suggest that there is room to improve commitment to early prevention.

Future research should focus on conducting longitudinal studies to better understand the causal relationships between socio-economic factors and oral health outcomes. Additionally, exploring the effectiveness of specific interventions aimed at improving access to affordable dental care and education for lower income populations will be crucial in addressing these disparities and improving overall oral health.

## 5. Conclusions

This study found that adolescents and young adults in Zagreb, Croatia, have relatively poor DMFT and OHIP-14 scores, indicating significant oral health problems and associated reductions in QoL, particularly among older participants and those of lower SES. The positive correlation between age and both DMFT and OHIP-14 scores emphasizes that oral health tends to deteriorate with age within this population. Our results also suggest that more invasive treatments, such as endodontic treatments and tooth extractions, are associated with poorer quality of life, underlining the importance of early prevention. Given these specific findings, future public health initiatives should focus on targeted preventive interventions tailored to adolescents and young adults, with an emphasis on early and regular dental visits, increased oral hygiene education, and interventions that address socio-economic inequalities.

## Figures and Tables

**Table 1:** Socio-economic status questionnaire.

Variables	Categories	
	Code	Name
Father’s education	1	lower and medium
	2	higher and high
Mother’s education	1	lower and medium
	2	higher and high
Father’s employment	1	other
	2	employed
Mother’s employment	1	other
	2	employed
Number of people in household	1	other
	2	with both parents and siblings
Number of siblings	0	none
	1	one
	2	two
	3	three or more
Type of housing	1	rental or other
	2	own apartment
	3	own house
Own room within household	1	shared with children or adults
	2	yes, own room
Number of cars in household	1	none
	2	one
	3	two or more
Vacations taken per year	1	none
	2	one
	3	two
	4	more than two
Financial condition of household	1	below average
	2	average
	3	well-off

**Table 2:** Distribution of socio-economic status (SES) of respondents by gender.

SES	Male N (%)	Female N (%)	Total N (%)
Low	26 (24.8)	32 (22.1)	58 (23.2)
Middle	56 (53.3)	83 (59.7)	139 (55.6)
High	23 (21.9)	30 (20.7)	53 (21.2)
Total	105 (42.0)	145 (58.0)	250 (100.0)

Pearson chi-square value = 0.400, df = 2, *p* = 0.819. 0 cells (0.0%) have expected count less than 5. The minimum expected count is 22.26.

**Table 3:** Distribution of respondents’ treatment by gender.

Treatment	Male N (%)	Female N (%)	Total N (%)
Prophylaxis	33 (31.4)	41 (28.3)	74 (29.6)
Restoration	55 (52.4)	80 (55.2)	135 (54.0)
Endodontics and extraction	17 (16.2)	24 (16.6)	41 (16.4)
Total	105 (42.0)	145 (58.0)	250 (100.0)

Pearson chi-square value = 0.297, df = 2, *p* = 0.862. 0 cells (0.0%) have expected count less than 5. The minimum expected count is 17.22.

**Table 4:** Distribution of treatment of respondents according to their socio-economic status (SES).

Treatment	Low N (%)	Middle N (%)	High N (%)	Total N (%)
Prophylaxis	17 (29.3)	44 (31.7)	13 (24.5)	74 (29.8)
Restoration	27 (46.6)	73 (52.5)	35 (66.0)	135 (54.0)
Endodontics and extraction	14 (24.1)	22 (15.8)	5 (9.4)	41 (16.4)
Total	58 (23.2)	139 (55.6)	53 (21.2)	250 (100.0)

Pearson chi-square value = 6.449, df = 4, *p* = 0.168. 0 cells (0.0%) have expected count less than 5. The minimum expected count is 8.69.

**Table 5:** Difference between the number of extracted teeth and the DMF index according to socio-economic status (SES).

Variables	SES	N	Mean	SD	F-Value	df_1_/df_2_	*p* *
DMFT	Low	58	8.09	5.56	2.548	2/247	0.080
	Middle	139	6.65	4.93			
	High	53	6.09	4.15			
	Total	250	6.87	4.97			
DMFT index	Low	58	25.27	17.39	2.548	2/247	0.080
	Middle	139	20.80	15.41			
	High	53	19.04	12.96			
	Total	250	21.46	15.52			

* One-way ANOVA test.

**Table 6:** Difference between the number of caries per person and the DMF index by gender.

Variable	Gender	N	Mean	SD	*t*-Value	df	*p* *
Caries	Male	105	2.76	3.07	2.804	248	0.009
	Female	145	1.85	2.08			
	Total	250	2.23	2.58			
DMFT	Male	105	6.77	5.50	0.261	248	0.794
	Female	145	6.94	4.56			
	Total	250	6.87	4.97			
DMFT index	Male	105	21.16	17.18	0.261	248	0.794
	Female	145	21.68	14.25			
	Total	250	21.46	15.52			

* Independent samples *t*-test.

**Table 7:** Difference between the number of healthy, carious, extracted, filled, and missing teeth per person and the DMFT index according to the treatment.

Number of Teeth	Treatment	N	Mean	SD	F-Value	df_1_/df_2_	*p* *
Healthy	Prophylaxis	74	25.03	3.25	28.494	2/247	<0.001
	Restoration	135	21.21	4.29			
	Endodontics and extraction	41	19.88	4.60			
	Total	250	22.12	4.49			
Carious	Prophylaxis	74	0.57	1.25	33.397	2/247	<0.001
	Restoration	135	2.61	2.36			
	Endodontics and extraction	41	4.00	3.32			
	Total	250	2.23	2.58			
Extracted	Prophylaxis	74	0.18	0.69	4.159	2/247	0.017
	Restoration	135	0.41	0.99			
	Endodontics and extraction	41	0.71	1.23			
	Total	250	0.39	0.97			
Filled	Prophylaxis	74	2.88	2.78	8.051	2/247	<0.001
	Restoration	135	4.81	3.73			
	Endodontics and extraction	41	4.85	3.80			
	Total	250	4.24	3.59			
DMFT	Prophylaxis	74	3.62	3.29	30.060	2/247	<0.001
	Restoration	135	7.83	4.60			
	Endodontics and extraction	41	9.56	5.72			
	Total	250	6.87	4.97			
DMFT index	Prophylaxis	74	11.32	10.29	30.060	2/247	<0.001
	Restoration	135	24.47	14.38			
	Endodontics and extraction	41	29.88	17.88			
	Total	250	21.46	15.52			

* One-way ANOVA test.

**Table 8:** Differences in OHIP by gender, SES, and treatment.

Gender	N	Mean	SD	*t*-Value	df	*p* *
Male	105	8.81	8.11	−1.979	248	0.049
Female	145	10.97	8.77			
Total	250	10.06	8.55			
**SES**	**N**	**Mean**	**SD**	**F-value**	**df_1/_df_2_**	*p* **
Low	58	12.67	10.81	3.656	2/247	0.027
Middle	139	9.14	7.21			
High	53	9.60	8.59			
Total	250	10.06	8.55			
**Treatment**	**N**	**Mean**	**SD**	**F-value**	**df_1/_df_2_**	*p* **
Prophylaxis	74	7.53	6.74	22.491	2/247	<0.001
Restoration	135	9.21	7.91			
Endodontics and extraction	41	17.44	10.21			
Total	250	10.06	8.55			

* Independent samples *t*-test; ** One-way ANOVA test.

## Data Availability

Dataset available on request from the authors.

## Abbreviations

The following abbreviations are used in this manuscript:

DMFT	Decayed, Missing, and Filled Teeth
OHIP	Oral Health Impact Profile
OHRQoL	Oral Health-Related Quality of Life
QoL	Quality of Life
SES	Socio-Economic Status
